# Evaluating Phthalate Contaminant Migration Using Thermal Desorption–Gas Chromatography–Mass Spectrometry (TD–GC–MS)

**DOI:** 10.3390/polym11040683

**Published:** 2019-04-15

**Authors:** Yukihiro Ouchi, Hiroyuki Yanagisawa, Shigehiko Fujimaki

**Affiliations:** Consumer & Retail Service Division, SGS Japan Inc., YBP East Tower 12F, 134 Godo-cho, Hodogaya-ku, Yokohama 240-0005, Japan; Yukihiro.Ouchi@sgs.com (Y.O.); hiroyuki.yanagisawa@sgs.com (H.Y.)

**Keywords:** phthalate migration, contaminant, thermal desorption, gas chromatography–mass spectrometry, additives, plasticizer, Fick’s law, solid/solid interface, plasticized PVC

## Abstract

This study describes a methodology for evaluating regulatory levels of phthalate contamination. By collecting experimental data on short-term phthalate migration using thermal desorption–gas chromatography–mass spectrometry (TD–GC–MS), the migration of di(2-ethylhexyl) phthalate (DEHP) from polyvinyl chloride (PVC) to polyethylene (PE) was found to be expressed by the Fickian approximation model, which was originally proposed for solid (PVC)/liquid (solvent) migration of phthalates. Consequently, good data correlation was obtained using the Fickian approximation model with a diffusion coefficient of 4.2 × 10^−12^ cm^2^/s for solid (PVC)/ solid (PE) migration of DEHP at 25 °C. Results showed that temporary contact with plasticized polymers under a normal, foreseeable condition may not pose an immediate risk of being contaminated by phthalates at regulatory levels. However, as phthalates are small organic molecules designed to be dispersed in a variety of polymers as plasticizers at a high compounding ratio, the risk of migration-related contamination can be high in comparison with other additives, especially under high temperatures. With these considerations in mind, the methodology for examining regulatory levels of phthalate contamination using TD–GC–MS has been successfully demonstrated from the viewpoint of its applicability to solid (PVC)/solid (PE) migration of phthalates.

## 1. Introduction

Plastic products are used extensively in our daily life and are indispensable materials for modern society. Several plastic products have been manufactured by adding various additives to petroleum-derived raw materials. For example, plasticizers soften plastics and facilitate the manufacturing of a variety of products [1]. However, plasticizers, such as phthalates, are regulated by global chemical laws as a type of hazardous substance that causes a threat to human health [2,3]. Certain phthalates are now classified as major hazardous substances that pose a threat to the reproductive system, causing such effects as birth defects or other problems related to reproductivity [4], and are suspected to have endocrine-disrupting properties. As a result, phthalates are being restricted in some areas, such as medical and childcare products [5], and even stricter controls are expected to be enforced on dibutyl phthalate (DBP), bis(butylbenzyl)phthalate (BBP), di(2-ethylhexyl) phthalate (DEHP), and diisobutyl phthalate (DIBP) [6] among others.

Phthalates are small organic molecules designed to disperse as plasticizers in various polymers without chemically bonding to the base polymer structures. Hence, phthalates can be migrated from one polymer to another [7]. Several commercially available soft polymers have been produced by intentionally adding phthalates at extremely high compound concentrations. For these soft polymers, the compounding ratio of phthalates reaches up to 30% or even higher. These soft polymers can be a source of phthalate contamination to the surrounding polymers. In addition, as listed in Table 1, most global laws set regulatory levels for phthalates as 0.1% (i.e., 1000 mg/kg).

Under such circumstances, contamination from phthalates via migration also possesses the risk of exceeding regulatory levels. Unfortunately, the impact of phthalate migration has often been overlooked when confirming compliance with chemical regulations. This study was conducted to address increasing concerns about phthalate contamination from the viewpoint of the recent worldwide ban on plasticizers.

To date, no study has closely examined the migration behavior of phthalates from the viewpoint of regulatory levels of contamination. This study aims to reveal several migration characteristics of phthalates associated with contamination at the regulatory level. With this in mind, gas chromatography–mass spectrometry (GC–MS) analysis was seen as a promising test method that is capable of detecting the migration of phthalates corresponding to the regulation level of 1000 mg/kg. It is essential to collect data repeatedly from samples to experimentally track the trend of migration. A test method requiring only a small amount of sample was therefore desirable to avoid any disturbance in ongoing migration tests. Thermal desorption (TD)–GC–MS was employed as the most suitable test method to evaluate phthalate contaminant migration [8,9,10] because it allows direct injection of samples with a minimum amount (e.g., 0.5 mg) for the detection of phthalates at regulatory levels.

## 2. Materials and Methods

An in-depth GC–MS analysis on the solid intersurface migration of phthalates was performed with the aim of clarifying issues related to its contamination.

### 2.1. Materials

The materials used included a blank (phthalate-free) polyethylene (PE) sheet and reference PE sheet containing seven types of phthalates (DIBP, DBP, BBP, DEHP, di-n-octyl phthalate (DNOP), diisononyl phthalate (DINP), and diisodecyl phthalate (DIDP)) (P/N: S225-31003-91, Shimadzu Corp., Kyoto, Japan) with a legal threshold of 1000 mg/kg and a joint part of a commercially available polyvinyl chloride (PVC) tube containing 7.4% DEHP. These materials were cut into small pieces (2 × 2 cm^2^) suitable for the migration test described below. In addition, the following reagent and material were used to collect supplemental data: ethanol (99.5%) (analytical grade, Kanto Chemical Co., Inc., Tokyo, Japan) and a commercially available insulation tape (polyethylene terephthalate) containing 22% (*w*/*w*) decabromodiphenyl ether (deca-BDE). Aluminum plates and foil with thickness 0.52 and 0.011 mm, respectively, were used as sealing tools.

### 2.2. Migration Test

**Figure 1:**
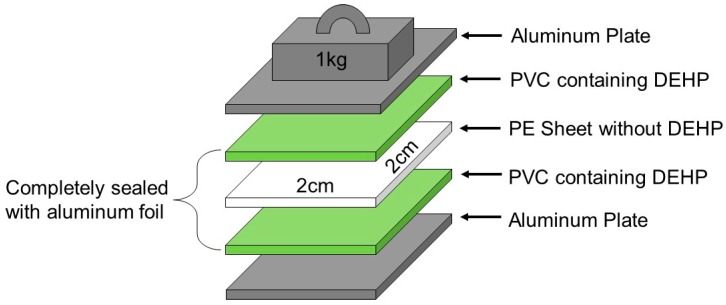
Sandwich system for the phthalate migration test. Legend: polyvinyl chloride (PVC), polyethylene (PE).

Figure 1 shows a schematic overview of the experimental setup used for the migration test. A blank PE sheet was placed as a sample between two PVC sheets (containing phthalates at 7.4% (w/w)) to form a sandwich configuration. The migration test was performed in reference to the ISO 177, which provides a standardized method for evaluating the tendency of plasticizers to migrate from plastic materials to other materials placed in close contact by monitoring the weight increase of the sample [11]. However, the gravimetric measurement set out in ISO 177 was not sensitive enough to incrementally evaluate time-sequential migration. Hence, a modified migration test was proposed specifically for this study using a highly sensitive GC–MS measurement. As a result, the experimental setup was scaled down to one-fifth of the size specified in ISO 177 while maintaining the same surface pressure. A small piece of PE blank sample (2 × 2 cm^2^) with a thickness of ~0.2 mm was placed between two PVC plates (2 × 2 cm^2^) with a thickness of 1.5 mm and kept under pressure (1.0 kg weight, Kanto Measure Co., Ltd., Kawasaki, Japan) at room temperature (25 °C) for 20 days. MEDI COOL (MPR-414F, manufactured by SANYO Electric Co., Ltd., Daito, Japan) and a vacuum drying oven DRV-420DA were used for the temperature-dependent migration test.

### 2.3. Monitoring of Phthalate Migration

The sample sandwiched between the PVCs was removed from the weight (1.0 kg) at appropriate time intervals in order to immediately measure the phthalate concentration. A small amount of the test sample was taken by a micropuncher (125D, 1.25 mm Φ, Frontier Laboratories Ltd., Koriyama, Japan) and weighed by an electronic balance (XS 105 DUV, Mettler Toledo International Inc., Columbus, OH, USA) to measure the change in concentration of the migrated phthalate. The sample was analyzed using GC–MS (GCMS-QP 2010 Ultra, Shimadzu Corp., Kyoto, Japan) with a TD unit (EGA/PY-3030D, Frontier Lab, Koriyama, Japan) mounted on top of the GC inlet. Here, the measured values were calibrated based on the actual sample weight. The uncertainty of measurement was confirmed to be 58 mg/kg for a DEHP standard reference material (1000 kg/mg). With regard to the reproducibility, a relative standard deviation (RSD) of 4.39% (n = 18) was confirmed for the standard reference material.

**Figure 2:**
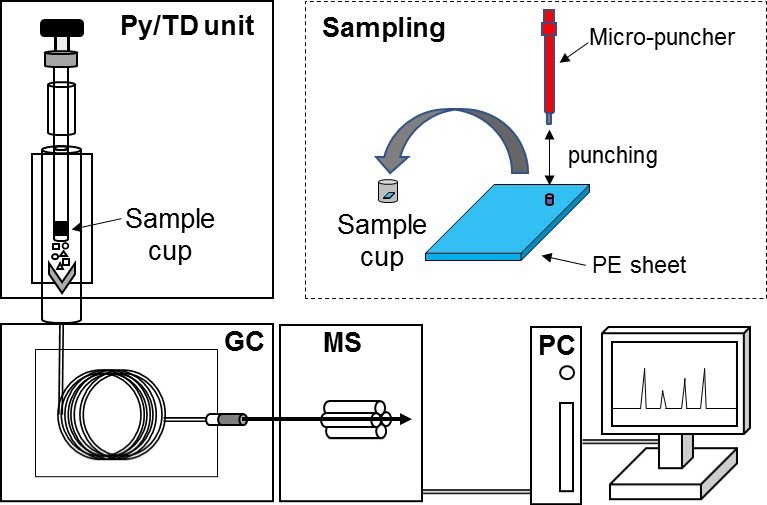
Schematic of the thermal desorption–gas chromatography–mass spectrometry (TD–GC–MS) system used.

Figure 2 shows a schematic diagram of the TD–GC–MS system. Samples weighing about 0.5 mg were placed in sample cups and then directly dropped into a furnace attached to the inlet of the GC unit for thermal desorption under a specific heat zone. The heating temperature was controlled at 300 °C to suppress the excessive decomposition of the base polymer. Thermally desorbed gaseous components were then transferred to the GC unit separated by a capillary column and detected with a mass spectrometer. The typical TD heating conditions and GC–MS parameter settings are listed in Table 2. A 15 m × 0.25 mm Inner Diameter (I.D.) GC column with a film thickness of 0.1 µm (Ultra ALLOY-5, 5% diphenyl dimethylpolysiloxane, Frontier Lab) was used for the measurement. The column temperature was increased from 80 °C to 300 °C at 20 °C/min and held at 300 °C for five minutes during the GC run. The injection mode was set to split with a ratio of 1/50, and helium (with a purity of greater than a volume fraction of 99.999%) was used as a carrier gas at a constant linear velocity of 52.6 cm/s. MS analysis was performed using a simultaneous switching function for scan and selected ion monitoring (SIM). Phthalates were identified based on their retention times and MS spectra. In MS quantitative analysis, the SIM signal was analyzed with a focus on phthalate-specific ions (see Table S1). The ion source was controlled at 230 °C with an interface temperature of 320 °C. For ionization, electron ionization (EI) was employed at 70 eV. The phthalate test method by TD–GC—MS has been standardized as part of the IEC62321 method through a sufficient verification process. Detailed validation data have been reported elsewhere [9,10,12].

A Fourier-transform infrared (FT-IR) spectrometer with an attenuated total reflectance (ATR) accessory was used in conjunction with TD–GC–MS to back up the monitoring of high-concentration phthalates compounded in polymers. Specifically, a Thermo Scientific Nicolet 380 FT-IR spectrometer and a Smart Orbit ATR accessory (Diamond ATR) were used to check for the presence or absence of plasticizers by running a library search and IR partial substructural analysis.

### 2.4. Migration Theory

The following Fickian approximation attracted our attention as one of the most appropriate models for time-sequential migration over a relatively short time frame [13,14]. The approximation is as follows:

M_t_/M_∞_ = 2(Dt/πl^2^)^1/2^ (1)

where M_t_ and M_∞_ are the total amount of migrated plasticizer at time t (s) and after infinite time, respectively. D (cm^2^/s) represents the diffusion coefficient, and l (cm) is the thickness of the PVC sheet.

Unfortunately, so far, the proposed migration model has been mostly reported in solid–liquid migration [13,14]. Still, this one-dimensional Fickian diffusion approximation is a good starting point for evaluating experimental data. At the same time, determining the applicability of the above Fickian approximation to solid intersurface migration was considered as one of the main challenges of this study.

## 3. Results

To date, migration of plasticizers from PVC to the surrounding medium has been studied under various conditions [13,15,16,17,18,19]. However, the diffusion of phthalates over a relatively short time frame has not yet been examined in detail in relation to a solid intersurface migration. This study dealt with PVC (plasticized)/solid (polymer) surface migration for the purpose of clarifying some migration characteristics of phthalates related to regulated level of contamination.

The migration behavior of phthalates was examined using a GC–MS equipped with a TD unit. At the same time, FT-IR was considered as an alternative to TD–GC–MS. Unfortunately, due to its lack of sensitivity and identification ability, FT-IR failed to track the migration of plasticizers at the regulatory level, as can be seen in the supplementary data (Figure S1).

TD–GC–MS was considered to be the most suitable instrument for direct analysis of migrated phthalates, and FT-IR was used only for collecting backup data. GC–MS measurement values were fitted to a diffusion equation based on the Fickian diffusion model for the interpretation and estimation of migration characteristics.

### 3.1. PVC (Plasticized)/Solid (Polymer) Intersurface DEHP Migration

**Figure 3:**
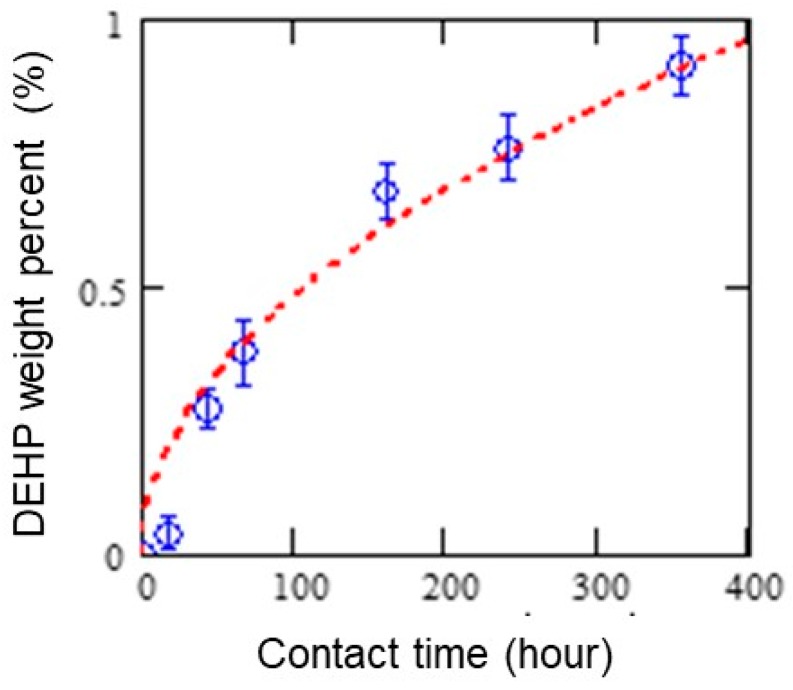
Time-dependent migration of DEHP from a PVC (7.4% (w/w)) to PE sheet. The error bars represent one standard deviation of the three measurements.

Figure 3 provides measurement values and a fitting curve for a PVC (plasticized)/solid (PE sheet) migration of DEHP over 400 h. The dashed line represents the estimated migration of phthalates derived from the equation described above.

**Figure 4:**
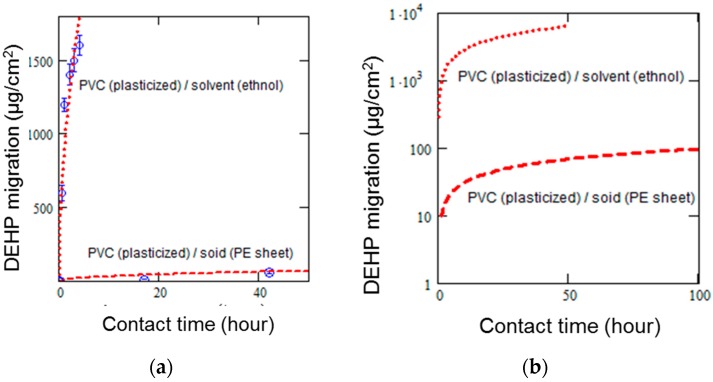
The difference in DEHP’s migration from PVC to solvent (ethanol) and solid (PE sheet); (**a**) comparison of DEHP’s migration and (**b**) extrapolated estimation of migration by the Fickian model. The error bars represent one standard deviation of the three measurements.

A good data correlation to the Fickian approximation model was obtained with 4.2 × 10^−12^ cm^2^/s of the diffusion coefficient. As mentioned earlier, the equation was initially proposed to express the PVC (plasticized)/liquid (solvent) migration with a diffusion coefficient of 2.72–10.1 × 10^−10^ cm^2^/s [13]. The difference in diffusion coefficient should be related to the coverage of the contact area between PVC and each contact medium. An additional experiment was performed to verify the migration of DEHP from PVC to different contact media. As shown in Figure 4a, the difference in DEHP’s migration from PVC to solvent (ethanol) and solid (PE sheet) was confirmed to be similar to that predicted from past literature [11]. Figure 4b provides extrapolated results by applying the migration model [13]. For reference, a schematic diagram of the experimental setup for testing DEHP’s migration from PVC (plasticized) to solvent (ethanol) is provided in the supplementary material (Figure S3). As a result, the Fickian approximation model can be considered applicable to the PVC (plasticized)/solid (PE) migration of DEHP given the good data fitting correlation and the reasonable diffusion coefficient. According to this result, the blank PE sheet reached the regulatory criterion (DEHP, 1000 mg/kg) after being in contact with PVC (plasticized) for about 20 h at room temperature.

### 3.2. Temperature-Dependent Migration Behavior of DEHP

**Figure 5:**
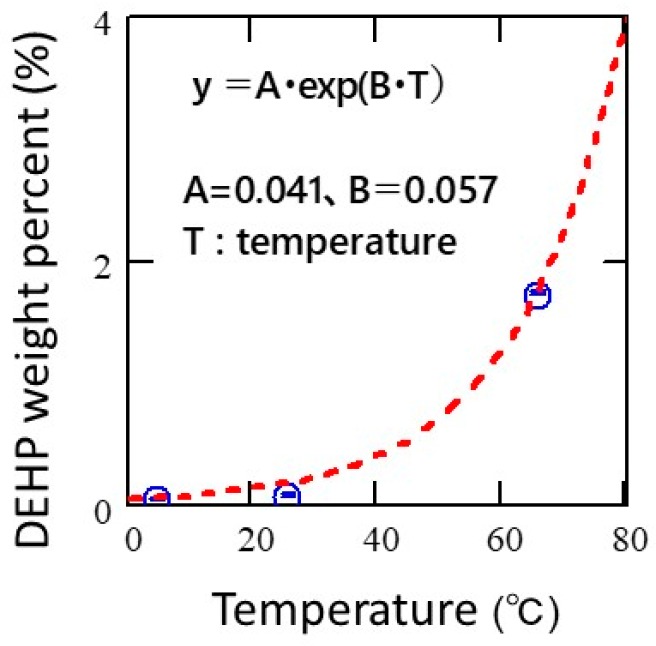
The change in DEHP weight percent due to temperature-dependent migration after 17 h of contact with plasticized PVC.

The temperature-dependent migration behavior of DEHP was examined in detail using TD–GC–MS. Figure 5 represents the change in DEHP weight percent due to temperature-dependent migration after 17 h of contact with plasticized PVC. As can be seen, the migration of DEHP exponentially increased with an increase in the temperature. The effects of temperature-dependent phthalate migration are discussed in detail later.

### 3.3. Comparison of Typical Plasticizer Migration

**Figure 6:**
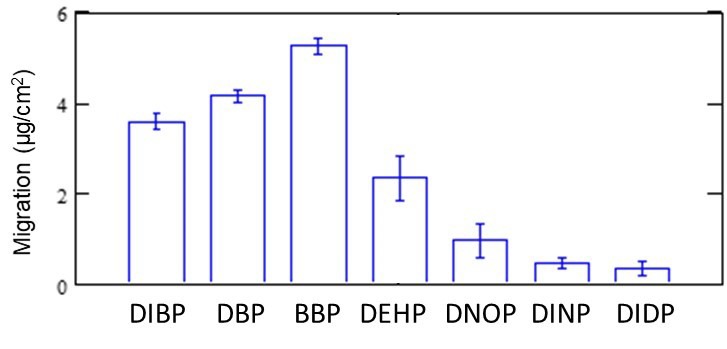
Differences in the migration of major phthalates. The error bars represent one standard deviation of the three measurements.

In addition, the migration behaviors, as shown in Figure 3 and Figure 4, are the result of DEHP in the PVC sheet. Migration behavior varies depending on the type of phthalate. Hence, migrations from a PE sheet containing seven types of phthalates (DIBP, DBP, BBP, DEHP, DNOP, DINP, and DIDP) at 1000 mg/kg were examined using TD–GC–MS. The concentration of the blank (phthalate-free) PE sheet after contacting the reference (1000 mg/kg for each phthalate) PE sheet at 25 °C for 21 h was measured by the same experimental method using TD–GC–MS as described above. Figure 6 shows the migration trend of each phthalate. Differences in migration rates were observed almost in accordance with the molecular weight and/or boiling point of each phthalate. In other words, the migration rate decreased in the order of molecular weight and boiling point.

## 4. Discussion

By collecting experimental data on short-term solid (PVC)/solid (PE) migration of phthalates using TD–GC–MS, the migration behavior was found to be able to be expressed with the Fickian approximation model, which was originally proposed for solid (PVC)/solvent (e.g., ethanol) diffusion in previous studies [13,14]. As a result, good data correlation with the Fickian approximation model was obtained with a diffusion coefficient of 4.2 × 10^−12^ cm^2^/s for solid (PVC)/ solid (PE) migration of DEHP at 25 °C. Obviously, the diffusion coefficient of DEHP for solid (PVC)/solid (PE) migration was estimated to be much smaller than that for solid (PVC)/solvent (ethanol) migration, and temporary or mild contact with plasticized PVC may not risk DEHP’s migration at regulatory levels. However, given that phthalates are small organic molecules designed to be dispersed in a variety of polymers as plasticizers at a high compounding ratio, the risk of migration-related contamination remains high compared to other additives. As shown in Figure 3, close contact of PVC/PE at 25 °C can cause DEHP’s migration over regulatory levels in ~20 h. As suggested in the supplementary data, there were no signs of migration in such a short time frame with other regulatory additives, such as brominated flame retardants (Deca-BDE) (see Tables S2 and S3). Moreover, it should be noted that DEHP’s migration drastically increased with an increase in temperature. Therefore, particular caution should be exercised when handling plasticized PVC at temperatures exceeding normal room temperature. In contrast, precautionary measures based on technical grounds should minimize the risk of plasticizer migration. Such phthalate migration can be restricted to below regulatory levels by avoiding hours of close contact with the contaminant at relatively high temperatures.

## 5. Conclusions

This study aimed to clarify the issues related to phthalate contamination using TD–GC–MS. GC–MS data were collected experimentally and fitted to a diffusion equation based on the Fickian diffusion model, which was originally proposed for the interpretation and estimation of DEHP’s PVC (plasticized)/solvent (e.g., ethanol) migration.

Good data correlation was obtained using the Fickian approximation model with a diffusion coefficient of 4.2 × 10^−12^ cm^2^/s for solid (PVC)/solid (PE) migration of DEHP at 25 °C. The diffusion coefficient of phthalate was found to be sufficiently small unless an organic solvent was present at the interface. With respect to the Fickian migration model, temporary contact with plasticized polymers under normal, foreseeable conditions may not pose an immediate risk of contamination by restricted phthalates at regulatory levels. However, considering that DEHP’s migration drastically increased with an increase in temperature, particular caution should be exercised when handling plasticized PVC at temperatures exceeding normal room temperature.

In conclusion, TD–GC–MS was proven to be effective in experimentally analyzing the migration behavior of phthalates. As mentioned above, the methodology for examining regulatory levels of phthalate contamination using TD–GC–MS has been successfully presented with respect to its applicability to PVC (plasticized)/solid (PE sheet) migration of DEHP.

## Figures and Tables

**Table 1:** Phthalate regulation in major chemical laws (legal thresholds: 1000 mg/kg).

Regulation	Phthalate Types
EU RoHS Directive (2011/65/EU)	DIBP, DBP, BBP, DEHP
REACH Annex XVII (EC) No.1907/2006	DBP, BBP, DEHP, DNOP, DINP, DIDP
CPSIA section 108	DIBP, DBP, BBP, DEHP, DINP, DPENP, DnHP, DCHP
Proposition 65 (California)	DBP, BBP, DEHP, DnHP, DINP, DIDP
EU Directive (2005/84/EC)	DBP, BBP, DEHP, DNOP, DINP, DIDP

Legend: di-n-octyl phthalate (DNOP), diisononyl phthalate (DINP), diisodecyl phthalate (DIDP), di-n-hexyl phthalate (DnHP), di-n-pentyl phthalate (DPENP), and dicyclohexyl phthalate (DCHP); EU RoHS: Restriction of Hazardous Substances (European); REACH: Registration, Evaluation, Authorization, and Restriction of Chemicals (European); CPSIA: Consumer Product Safety Improvement Act (United States).

**Table 2:** TD heating conditions and GC–MS parameter settings.

Apparatus	Parameters	Settings
Furnace (single shot analysis)	Furnace temperature	300 °C constant
GC	Interface temperature	300 °C
	Sampling time	1 min
	Column	5% diphenyl dimethylpolysiloxane; length: 15 m; ID: 0.25 mm; film thickness: 0.1 μm
	Injection port temperature	320 °C
	Column oven temperature	80 °C→(20 °C/min)→300 °C (5 min)
	Injection mode	Split (split ratio, 1/50)
	Carrier gas	Helium, 52.1 cm/s, constant linear velocity
MS	Ion source temperature	230 °C
	Ionization method	Electron ionization, 70 eV

## Supplementary Materials

The following are available online at https://www.mdpi.com/2073-4360/11/4/683/s1. Table S1: List of quantitative and confirmation ions. Table S2: Thermal desorption (TD) heating conditions and gas chromatography–mass spectrometry (GC–MS) parameter settings for Deca-BDE. Table S3: Concentration of Deca-BDE in the sample after 21 h of contact with a PET sheet containing 22% (w/w) Deca-BDE. Figure S1: FT-IR spectra of various polymers. (a) PE sheet after 352 h of contact with a PVC sheet containing 7.4% (w/w) DEHP; (b) PVC containing 7.4% (w/w) DEHP; (c) standard DEHP spectrum retrieved from OMNIC 7.3 library. FT-IR was not sensitive enough to detect DEHP’s migration at the regulatory level. Figure S2: Schematic of DEHP migration test from PVC (plasticized) to solvent (ethanol).

## References

[1] Orecchio S., Indelicato R., Barreca S. (2014). Determination of selected phthalates by gas chromatography–mass spectrometry in mural paintings from palermo (Italy). Microchem. J..

[2] (2018). Standard Operating Procedure for Determination of Phthalates CPSC-CH-C1001-09.4.

[3] Commission of the European Communities Amending Annex XVII to regulation (EC) No 1907/2006 of the European Parliament and of the Council Concerning the Registration. https://eur-lex.europa.eu/legal-content/EN/TXT/?uri=uriserv:OJ.L_.2006.396.01.0001.01.ENG.

[4] (2003). Comment from the union participant in the IARC Working Group that downgraded DEHP. Int. J. Occup. Environ. Health.

[5] Veeramachaneni D.N.R., Furr J., Ostby J., Gray L.E., Parks L., Price M. (2000). Perinatal exposure to the phthalates DEHP, BBP, and DINP, but not DEP, DMP, or DOTP, alters sexual differentiation of the male rat. Toxicol. Sci..

[6] (2011). Directive 2011/65/EU of the European Parliament and of the Council of 8 June 2011 on the Restriction of the Use of Certain Hazardous Substances in Electrical and Electronic Equipment Text with EEA Relevance. https://eur-lex.europa.eu/legal-content/EN/TXT/?uri=celex%3A32011L0065.

[7] Ambrogi V., Brostow W., Carfagna C., Pannico M., Persico P. (2012). Plasticizer migration from cross-linked flexible PVC effects on tribology and hardness. Polym. Eng. Sci..

[8] Earls A.O., Axford I.P., Braybrook J.H. (2003). Gas chromatography–mass spectrometry determination of the migration of phthalate plasticisers from polyvinyl chloride toys and childcare articles. J. Chromatogr. A.

[9] Guart A., Bono-Blay F., Borrell A., Lacorte S. (2011). Migration of plasticizers phthalates, bisphenol a and alkylphenols from plastic containers and evaluation of risk. Food Addit. Contam..

[10] Maruyama F., Fujimaki S., Sakamoto Y., Kudo Y., Miyagawa H. (2015). Screening of phthalates in polymer materials by pyrolysis GC/MS. Anal. Sci..

[11] Yanagisawa H., Kudo Y., Nakagawa K., Miyagawa H., Maruyama F., Fujimaki S. (2018). Simultaneous screening of major flame retardants and plasticizers in polymer materials using pyrolyzer/thermal desorption gas chromatography mass spectrometry (PY/TD-GC-MS). Molecules.

[12] Yanagisawa H., Maruyama F., Fujimaki S. (2019). Verification of simultaneous screening for major restricted additives in polymer materials using pyrolyzer/thermal desorption gas–chromatography mass spectrometry (PY/TD-GC-MS). J. Anal. Appl. Pyrolysis.

[13] Determination of Certain Substances in Electrotechnical Products Determination of Certain Substances in Electrotechnical Products. Part 8. International Electrotechnical Commission Technical Committee 111 2017. https://webstore.iec.ch/publication/32719.

[14] Kim J.H., Kim S.H., Lee C.H., Nah J.W., Hahn A. (2003). DEHP migration behavior from excessively plasticized PVC sheets. Bull. Korean Chem. Soc..

[15] Papaspyrides C.D., Duvis T. (1990). Loss of plasticizers from polymer films to liquid environments: Counterdiffusion aspects versus immersion temperature and ultra-violet-induced surface crosslinking. Polymer.

[16] Al-Natsheh M., Alawi M., Fayyad M., Tarawneh I. (2015). Simultaneous GC–MS determination of eight phthalates in total and migrated portions of plasticized polymeric toys and childcare articles. J. Chromatogr. B.

[17] Ekelund M., Azhdar B., Hedenqvist M.S., Gedde U.W. (2008). Long-term performance of poly(vinyl chloride) cables, part 2: Migration of plasticizer. Polym. Degrad. Stab..

[18] Kampouris E.M., Regas F., Rokotas S., Polychronakis S., Pantazoglou M. (1975). Migration of PVC plasticizers into alcohols. Polymer.

[19] Steiner I., Scharf L., Fiala F., Washuttl J. (1998). Migration of di-(2-ethylhexyl) phthalate from PVC child articles into saliva and saliva simulant. Food Addit. Contam..

