# Supplementary material for: Evaluating Phthalate Contaminant Migration Using Thermal Desorption–Gas Chromatography–Mass Spectrometry (TD–GC–MS)
Source: Polymers (Basel). 2019 Apr 15;11(4):683. doi: 10.3390/polym11040683 (PMC6523191; doi:10.3390/polym11040683)
Supplement: Supplementary file 1 [file polymers-11-00683-s001.pdf]

polymers

Supplementary material

Evaluating phthalate contaminants migration using thermal desorption–gas chromatography–mass spectrometry (TD-GC-MS)

Yukihiro Ouchi, Hiroyuki Yanagisawa and Shigehiko Fujimaki *

Consumer & Retail Service Division, SGS Japan Inc. YBP East Tower 12F, 134 Godo-cho, Hodogaya-ku, Yokohama 240-0005, Japan; Yukihiro.Ouchi@sgs.com (Y.O.); hiroyuki.yanagisawa@sgs.com (H.Y.)

* Correspondence: shigehiko.fujimaki@sgs.com; Tel.: +81-45-330-1101

Table S1 quantitative and confirmation ions

	Quantitative ion (*m/z*)	Confirmation ion (*m/z*)
DIBP	223	205
DBP	223	205
BBP	206	91
DEHP	279	167
DNOP	279	167
DINP	293	167
DIDP	307	167

Table S2 Thermal desorption (TD) heating conditions and gas chromatography–mass spectrometry (GC–MS) parameter settings for Deca-BDE.

Apparatus	Parameters	Settings
Furnace (Heart-cut analysis)	Furnace temperature	200 °C → 40 °C/min → 300 °C → 10 °C/min → 340 °C (1 min)
	Interface temperature	300 °C
	Sampling time	7.5 min
GC	Column	5% diphenyl dimethylpolysiloxane; length: 15 m; I.D.: 0.25 mm; film thickness: 0.1 µm
	Injection port temperature	320 °C
	Column oven temperature	100 °C → (20 °C/min) → 320 °C (5 min)
	Injection mode	Split (split ratio: 1/50)
	Carrier gas	Helium, 52.1 cm/s, constant linear velocity
MS	Ion source temperature	230 °C
	Ionization method	Electron ionization (EI); 70 eV

Table S3 Deca-BDE migration in the sample after 21 hours of contact with a PET sheet containing 22% (w / w) Deca-BDE.

Unit (mg/cm^2)	n=1	n=2	n=3	Average	STDEV
Daca-BDE after 21 hours of contact	1.8×10^{-3}	3.6×10^{-3}	3.6×10^{-3}	3.1×10^{-3}	1.1×10^{-3}

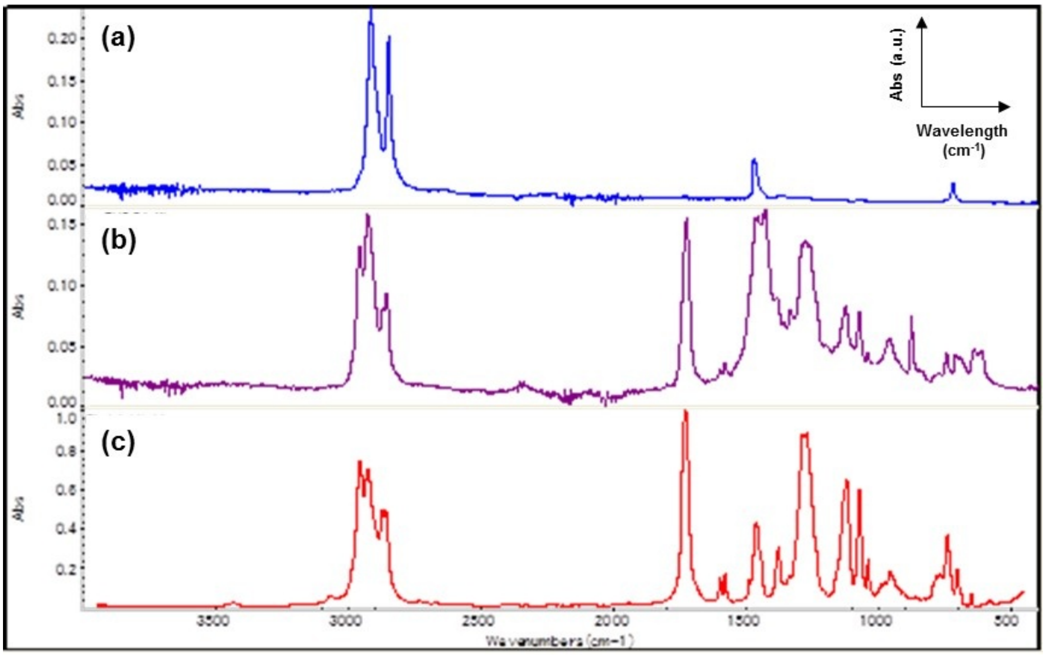

Fig. S1 FT-IR spectra of various polymers. (a) PE sheet after 352 hours of contact with a PVC sheet containing 7.4% (w/w) DEHP; (b) PVC containing 7.4% (w/w) DEHP; (c) Standard DEHP spectrum retrieved from OMNIC 7.3 library. FT-IR was not sensitive enough to detect the DEHP migration at the regulatory level.

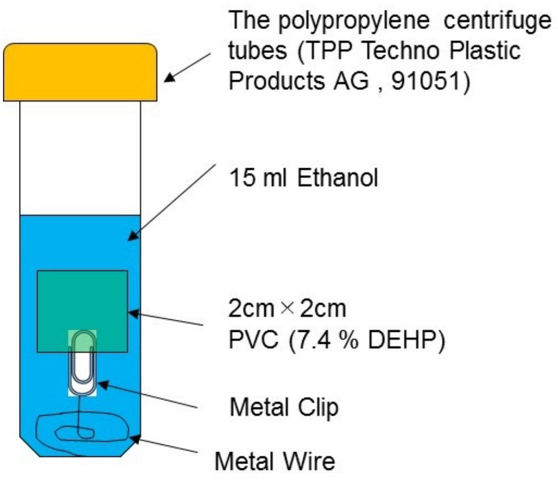

Fig.S2 Schematic of DEHP’s migration test from PVC (plasticized) to solvent (ethanol).
